# Adaptations to the British Society of Gastroenterology guidelines on the management of acute severe UC in the context of the COVID-19 pandemic: a RAND appropriateness panel

**DOI:** 10.1136/gutjnl-2020-321927

**Published:** 2020-06-08

**Authors:** Shahida Din, Alexandra Kent, Richard C Pollok, Susanna Meade, Nicholas A Kennedy, Ian Arnott, R Mark Beattie, Felix Chua, Rachel Cooney, Robin J Dart, James Galloway, Daniel R Gaya, Subrata Ghosh, Mark Griffiths, Laura Hancock, Richard Hansen, Ailsa Hart, Christopher Andrew Lamb, Charlie W Lees, Jimmy K Limdi, James O Lindsay, Kamal Patel, Nick Powell, Charles D Murray, Chris Probert, Tim Raine, Christian Selinger, Shaji Sebastian, Philip J Smith, Phil Tozer, Andrew Ustianowski, Lisa Younge, Mark A Samaan, Peter M Irving

**Affiliations:** 1 Department of Gastroenterology, Western General Hospital, Edinburgh, UK; 2 Gastroenterology and Hepatology Unit, University of Edinburgh, Edinburgh, UK; 3 Department of Gastroenterology, King’s College Hospital NHS Foundation Trust, London, UK; 4 Faculty of Life Sciences & Medicine, King's College London, London, UK; 5 Department of Gastroenterology, St George's Healthcare NHS Trust, London, UK; 6 Institute of Infection and Immunity, St George's University Hospitals NHS Foundation Trust, London, UK; 7 Department of Gastroenterology, Guy's and Saint Thomas' Hospitals NHS Trust, London, UK; 8 Department of Gastroenterology, Royal Devon and Exeter NHS Foundation Trust, Exeter, UK; 9 Exeter IBD Research Group, University of Exeter, Exeter, UK; 10 Department of Paediatric Gastroenterology, Southampton Children's Hospital, Southampton, UK; 11 Interstitial Lung Disease Unit, Department of Respiratory Medicine, Royal Brompton Hospital, London, UK; 12 Department of Gastroenterology, Queen Elizabeth Hospital Birmingham, Birmingham, UK; 13 Department of Gastroenterology, Royal Free Hospital, London, UK; 14 Department of Rheumatology, King's College Hospital, London, UK; 15 Gastroenterology Unit, Glasgow Royal Infirmary, Glasgow, UK; 16 Peri-operative Medicine, Barts Health NHS Trust, London, UK; 17 Faculty of Medicine, National Heart and Lung Institute, London, UK; 18 Department of General Surgery, Manchester University NHS Foundation Trust, Manchester, UK; 19 Paediatric Gastroenterology and Nutrition, Royal Hospital for Children, Glasgow, UK; 20 IBD Unit, St Mark's Hospital, London, UK; 21 Antigen Presentation Research Group, Imperial College London, London, UK; 22 Institute of Cellular Medicine, Newcastle University, Newcastle upon Tyne, UK; 23 Department of Gastroenterology, Newcastle upon Tyne Hospitals NHS Foundation Trust, Newcastle upon Tyne, UK; 24 Centre for Genomic and Experimental Medicine, University of Edinburgh, Edinburgh, UK; 25 Department of Gastroenterology, Pennine Acute Hospitals NHS Trust, Manchester, UK; 26 Manchester Academic Health Science Centre, Manchester, UK; 27 Department of Gastroenterology, Barts Health NHS Trust, London, UK; 28 Division of Digestive Diseases, Imperial College London, London, UK; 29 Gastroenterology Research Unit, Department of Cellular and Molecular Physiology, University of Liverpool Institute of Translational Medicine, Liverpool, UK; 30 Department of Gastroenterology, Cambridge University Hospitals NHS Foundation Trust, Cambridge, UK; 31 Leeds Gastroenterology Institute, Leeds Teaching Hospitals NHS Trust, Leeds, UK; 32 Department of Gastroenterology, Hull University Teaching Hospitals NHS Trust, Hull, UK; 33 Department of Immunuology and Inflammation, Hull York Medical School, Hull, Kingston upon Hull, UK; 34 Department of Gastroenterology, Royal Liverpool University Hospital, Liverpool, UK; 35 Department of Infectious Disease, North Manchester General Hospital, Manchester, UK; 36 Crohn's and Colitis UK, Saint Albans, UK; 37 Peter Gorer Department of Immunobiology, School of Immunology and Microbial Sciences, King's College London, London, UK

**Keywords:** ulcerative colitis, clinical decision making, IBD clinical

## Abstract

**Objective:**

Management of acute severe UC (ASUC) during the novel COVID-19 pandemic presents significant dilemmas. We aimed to provide COVID-19-specific guidance using current British Society of Gastroenterology (BSG) guidelines as a reference point.

**Design:**

We convened a RAND appropriateness panel comprising 14 gastroenterologists and an IBD nurse consultant supplemented by surgical and COVID-19 experts. Panellists rated the appropriateness of interventions for ASUC in the context of severe acute respiratory syndrome coronavirus-2 (SARS-CoV-2) infection. Median scores and disagreement index (DI) were calculated. Results were discussed at a moderated meeting prior to a second survey.

**Results:**

Panellists recommended that patients with ASUC should be isolated throughout their hospital stay and should have a SARS-CoV-2 swab performed on admission. Patients with a positive swab should be discussed with COVID-19 specialists. As per BSG guidance, intravenous hydrocortisone was considered appropriate as initial management; only in patients with COVID-19 pneumonia was its use deemed uncertain. In patients requiring rescue therapy, infliximab with continuing steroids was recommended. Delaying colectomy because of COVID-19 was deemed inappropriate. Steroid tapering as per BSG guidance was deemed appropriate for all patients apart from those with COVID-19 pneumonia in whom a 4–6 week taper was preferred. Post-ASUC maintenance therapy was dependent on SARS-CoV-2 status but, in general, biologics were more likely to be deemed appropriate than azathioprine or tofacitinib. Panellists deemed prophylactic anticoagulation postdischarge to be appropriate in patients with a positive SARS-CoV-2 swab.

**Conclusion:**

We have suggested COVID-19-specific adaptations to the BSG ASUC guideline using a RAND panel.

**Significance of this study**

**What is already known on this subject?**

- The British Society of Gastroenterology (BSG) has published evidence-based guidelines for the management of patients with acute severe UC (ASUC), but it is unknown whether these are appropriate in the setting of severe acute respiratory syndrome coronavirus-2 (SARS-CoV-2) infection.
- Currently there are limited data to inform clinicians in this area and there is no published guidance for the management of ASUC in the setting of the COVID-19 pandemic.

**What are the new findings?**

- The current BSG IBD guidelines provide a management pathway which remains largely appropriate during the COVID-19 pandemic.
- However, some treatment options were deemed uncertain or inappropriate in patients with established COVID-19 pneumonia.
- It is appropriate to involve COVID-19 specialists in decision-making for patients with ASUC who are SARS-CoV-2 positive.
- Steroid tapering as per BSG guidance was deemed appropriate for all patients apart from those with COVID-19 pneumonia in whom a 4–6 week taper was preferred.
- Prophylactic anticoagulation postdischarge is appropriate in patients with a positive SARS-CoV-2 swab.

**Significance of this study**

**How might it impact on clinical practice in the foreseeable future?**

- This paper summarises available evidence and provides expert opinion for the appropriate management of patients with ASUC during the COVID-19 pandemic.
- It also highlights areas of uncertainty which may help direct areas of future research.

## Introduction

The novel coronavirus severe acute respiratory syndrome coronavirus-2 (SARS-CoV-2) was first reported in December 2019 and its spread led to the declaration of a pandemic by the WHO on 11 March 2020. Infection varies in severity from asymptomatic carriage to an acute respiratory illness which, at its most severe, results in acute respiratory distress syndrome (ARDS) with hyperinflammation and cytokine storm syndrome.^1^ By mid-May 2020, there have been nearly 5 million cases reported worldwide with over 300 000 deaths.^2^ Risk factors associated with more severe COVID-19 include older age, male sex, hypertension, cardiovascular disease, respiratory disease, diabetes, renal failure and ethnicity.^3^ Neither an effective medical therapy nor a vaccine has yet been described, although numerous candidates are under evaluation.

Acute severe UC (ASUC) occurs in up to 25% of patients with UC and is associated with a mortality of approximately 1%.^4 5^ The management of ASUC is particularly challenging in the context of SARS-CoV-2 as the typical presenting features of ASUC, namely diarrhoea with raised inflammatory markers, often in association with a fever, may mimic those of COVID-19. ASUC is managed with high-dose parenteral corticosteroids, progressing to rescue therapy and/or surgery in those who fail to respond adequately.^6^ The safety of all these interventions in the context of COVID-19 is unclear. For example, there are concerns that corticosteroids may increase the risk of acquiring SARS-CoV-2 infection and/or worsen the severity of COVID-19.^7^ In addition, the commonly used rescue therapies, infliximab and ciclosporin are associated with an increased risk of infection, particularly if used in combination with immunomodulators such as thiopurines or steroids.^8^ Finally, individuals in whom corticosteroids and rescue therapy fail require urgent colectomy which is associated with high morbidity and mortality in patients infected with SARS-CoV-2.^9^ However, withholding treatment in ASUC is clearly not an option in view of the high mortality (in excess of 20%) associated with such an approach.^10^

While national and international registries continue to collate data regarding patients with IBD with COVID-19, very few cases relate to the management of ASUC. The PREPARE IBD (physician response to disease flares and patient adaptation in response to events in IBD during theCOVID-19 pandemic) study (www.prepareibd.org) is collecting data from patients with IBD who are admitted to hospital during the pandemic, as well as from those who develop confirmed or suspected SARS-CoV-2 infection. As of 8 May 2020, 19 patients with severe active UC including four with suspected or confirmed COVID-19 had been identified (S Sebastian, personal communication, 2020). The Surveillance Epidemiology of Coronavirus Under Research Exclusion-IBD registry (https://covidibd.org/) is collating data on patients with IBD with confirmed coronavirus, with 1074 patients included to date, the majority of whom have Crohn’s disease; details of how many in the cohort have ASUC are not yet available.^11^ Finally, in case series from Italy and Spain, 4 of 79 and 1 of 40 patients, respectively, had COVID-19 in conjunction with ASUC^12 13^ (the number of patients with ASUC in the Italian case series was provided on request from authors).

Treatment of ASUC during the COVID-19 pandemic presents substantial management dilemmas in the absence of a high-quality evidence base to guide clinicians. We therefore aimed to address this deficit of informed guidance by convening a RAND appropriateness panel. Current British Society of Gastroenterology (BSG) guidelines were used as a reference point to highlight differences to current management.^6^

## Methods

### Study overview

The RAND/UCLA (University of California, Los Angeles) appropriateness method uses a modified Delphi panel approach and combines expert opinion with the best available evidence to determine the appropriateness of specific practices in certain clinical situations.^14^ It is particularly useful in areas of uncertainty in which evidence is insufficient to guide day-to-day clinical practice, such as in the COVID-19 pandemic.^15^

The aim of this RAND panel was to provide clarity on the management of ASUC, as defined by Truelove and Witts criteria, in the context of the COVID-19 pandemic.^10^ The panel sought to identify areas where it was appropriate to deviate from current BSG ASUC guidance and consider alternative strategies.

We assembled a 15-person panel comprising representatives from the BSG IBD Section Committee, the BSG IBD Clinical Research Group (CRG) and other gastroenterologists, each from different IBD centres across the UK, as well as an IBD nurse consultant (online supplementary table 1). A web-based questionnaire was created and iteratively improved before being completed by all panellists prior to a moderated online meeting. We circulated a list of relevant publications with the questionnaire, comprising the current BSG guidelines on the management of ASUC^6^ along with up-to-date publications about COVID-19 in general and specifically in relation to IBD. Due to the rapid growth of available data, the panel used a range of instant messaging services to disseminate publications that were not available at the time of the initial literature review.

10.1136/gutjnl-2020-321927.supp1 Supplementary data

Panellists rated the appropriateness of management options at five different time points during the course of admission for ASUC (admission, first-line therapy, rescue therapy, continued medical therapy and surgery) in the context of absence of, or varying severity of SARS-CoV-2 infection. They were asked to grade the appropriateness of specific interventions on a scale of 1–9 (where 1–3 is inappropriate, 4–6 is uncertain and 7–9 is appropriate). The responses were summarised and anonymised before being presented at a virtual meeting in May 2020 with the aim of allowing discussion which ensured a common understanding of the questions and which focused on areas of disagreement, without trying to force consensus. Also present at the meeting were non-voting specialists who provided expert opinion with regards to IBD surgery (PT, LH), rheumatology (JG), intensive care (MG), respiratory medicine (FC) and infectious diseases (AU). In practice, several specialities may provide expert opinion in COVID-19 management, including intensivists, respiratory physicians and infectious disease physicians. We, therefore, used the encompassing term ‘COVID-19 specialist’ to represent this group. Finally, the Chairs of the BSG IBD Section Committee (IA) and the BSG IBD CRG (CAL) were also present. The moderators (PMI, MAS) neither expressed opinions on management nor voted, but were experts both in RAND panels and in the management of IBD. After the meeting, a second online survey comprising 91 questions, which had been slightly modified from the initial questionnaire following discussion at the meeting, was circulated for completion.

Several assumptions were made for clarity. First, patients were assumed to have a confirmed diagnosis of UC with intercurrent gastrointestinal infection having been excluded. Second, if this was not an index presentation, patients were assumed to have received optimised 5-aminosalicylic acid therapy prior to admission and were also presumed to be biological-naive. In addition, where ciclosporin was suggested as an option, it was assumed that the patient was thiopurine-naive. Third, other than those areas addressed in the survey, the management of ASUC was assumed to be in line with BSG guidance.^6^ Finally, where steroid weaning or discontinuation was considered, it was assumed that patients could safely stop steroids without the risk of Addisonian crisis.

In addition, in the section about first-line medical therapy, panellists assumed patients were not steroid refractory. For the rescue therapy section, patients were assumed to have ongoing ASUC despite 3 days of intravenous corticosteroid therapy and had reached standard criteria for rescue therapy.^16^ For the continuing medical therapy section, patients were assumed to have responded to intravenous corticosteroids sufficiently to switch to oral prednisolone and were ready to be discharged from hospital. Lastly, as per RAND methodology, respondents were advised to make decisions without considering local availability of treatments or cost.

### Analysis

For each scenario, median scores were calculated with a score of <3.5 being considered inappropriate, ≥3.5 but <6.5 uncertain and ≥6.5 appropriate. We used the validated RAND disagreement index (DI) to define disagreement among panellists using the equation outlined below.^14^ A DI ≥1 denotes disagreement. Any scenario in which disagreement was found was scored as uncertain, regardless of the median score.

$$DI=\frac{70\%ile-30\%ile}{2.35+\left(1.5\times abs\left(5-\frac{70\%ile+30\%ile}{2}\right)\right)}$$

## Results

### Overall results

**Figure 1:**
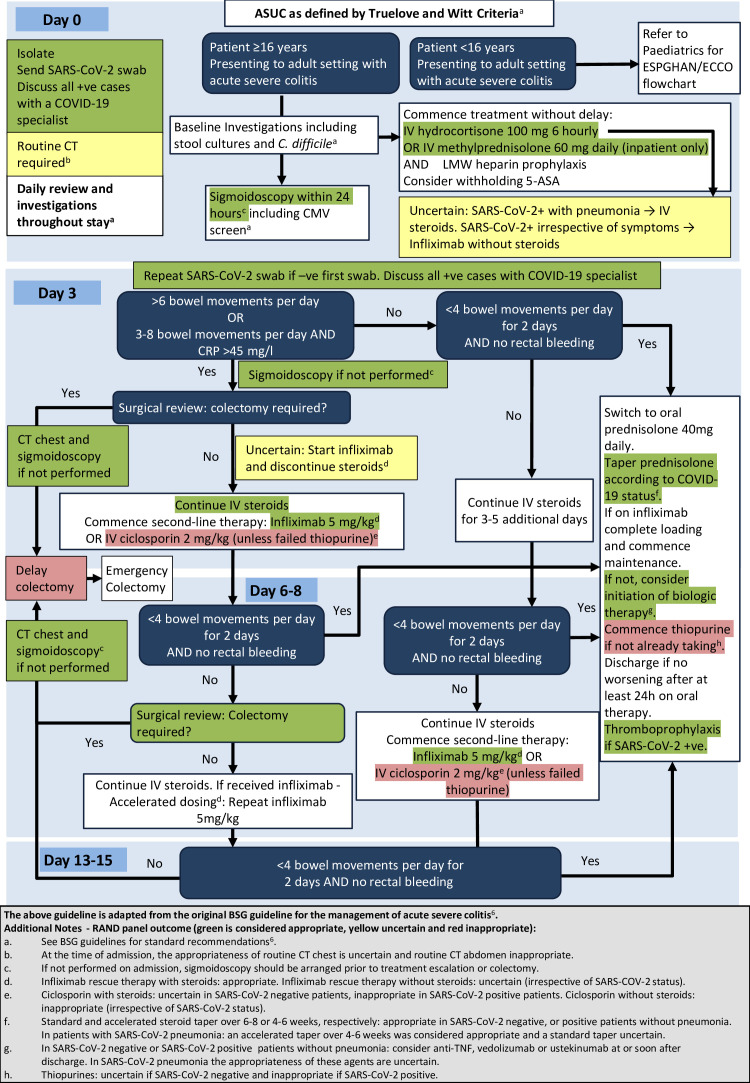
5-Adaptations to the BSG guideline for the management of ASUC in the context of COVID-19. ASA, 5-aminosalicylic acid; ASUC, acute severe UC; BSG, British Society of Gastroenterology; *C.difficle*, *Clostridium difficile*; CMV, cytomegalovirus; CRP, C reactive protein; ECCO, European Crohn's and Colitis Organisation; ESPGHAN, European Soceity for Paediatric Gastroenterology, Hepatology and Nutrition; IV, intravenous; LMW, low molecular weight; SARS-CoV-2, severe acute respiratory syndrome coronavirus-2; TNF, tumour necrosis factor.

Of the 91 clinical scenarios, panellists rated 28 as appropriate, 19 as uncertain and 44 as inappropriate. After the second round of voting, agreement was present for all scenarios (DI<1). The key findings are summarised below and their relationship to current BSG guidance is highlighted in figure 1. A detailed list of all scenarios, complete with median score, appropriateness rating and DI can be found in online supplementary table 2.

10.1136/gutjnl-2020-321927.supp2 Supplementary data

### Indications for investigations, inpatient isolation and specialist referral

The panellists agreed that all patients admitted to hospital with ASUC should have a SARS-CoV-2 swab performed on admission. If the result was negative it was deemed appropriate to repeat the swab at the point of requiring rescue therapy and/or surgery to exclude subclinical infection. It was also considered appropriate to isolate all patients throughout their hospital stay, irrespective of their COVID-19 status (table 1).

**Table 1:** Appropriateness of patient isolation and investigation in patients admitted with acute severe UC in the context of the COVID-19 pandemic

	On admission	Prior to rescue therapy	Prior to colectomy
Inpatient isolation	All patients		
SARS-CoV-2 swab	Performed in all patients	Repeat swab if initial swab negative	Repeat swab if initial swab negative
Flexible sigmoidoscopy	≤24-hour admission	If not performed	If not performed
		If already performed	If already performed
CT chest	Performed in all patients		Performed in all patients
CT abdomen and pelvis	Performed in all patients		

Green is considered appropriate, yellow uncertain and red inappropriate.

SARS-CoV-2, severe acute respiratory syndrome coronavirus-2.

It was rated appropriate to perform a flexible sigmoidoscopy within 24 hours of admission. If a patient had not had a flexible sigmoidoscopy on admission, it was considered appropriate that one should be performed prior to rescue therapy or colectomy. Repeating this test at these time points was deemed unnecessary in patients who had already had a flexible sigmoidoscopy performed.

Routine CT scanning of the abdomen/pelvis on admission (in addition to abdominal X-ray) was deemed inappropriate. However, the appropriateness of routine chest CT on admission was rated as uncertain. The one scenario in which a CT scan of the chest was felt to be appropriate for all patients irrespective of COVID-19 status was in the context of patients requiring colectomy.

Throughout the scenarios, the panellists considered the appropriateness of discussion with COVID-19 specialists. In patients without symptoms or signs of COVID-19 and with a negative swab, this was deemed inappropriate if receiving first-line therapy but uncertain in patients requiring rescue therapy. However, it was considered appropriate in all patients with a positive swab, irrespective of the presence of symptoms or signs of COVID-19.

### Initial treatment of ASUC

As per BSG guidance, intravenous hydrocortisone, 100 mg, four times per day (or equivalent) was rated appropriate as the initial management of patients presenting with ASUC in the absence of symptoms and signs of COVID-19 pneumonia. In patients with COVID-19 pneumonia, use of hydrocortisone was deemed uncertain. Other possible treatments (poorly bioavailable oral steroids, for example, budesonide multimatrix and beclometasone modified release, infliximab either with or without steroids, ciclosporin or tofacitinib) were considered inappropriate. The exception was infliximab (without steroids) which was considered uncertain in patients with a positive swab for SARS-CoV-2, either with or without signs of COVID-19 pneumonia. Ambulatory outpatient management with daily intravenous methylprednisolone was rated as inappropriate in all patients with ASUC regardless of SARS-CoV-2 status, as was management by immediate colectomy unless complications mandating emergency surgery were present such as toxic megacolon, perforation or severe haemorrhage (table 2).

**Table 2:** Appropriateness of treatment options in acute severe UC in the context of the COVID-19 pandemic: first-line medical therapy

	First-line medical therapy				
Negative SARS-CoV-2 swab WITHOUT respiratory symptoms	Inpatient intravenous steroids*	Poorly bioavailable steroids†	IFX alone	Tofacitinib	Discussion with COVID-19 specialist‡
	Ambulatory intravenous steroids§	Intravenous steroids*+IFX	Ciclosporin	Colectomy	
Positive SARS-CoV-2 swab WITHOUT respiratory symptoms or signs of COVID-19 pneumonia	Inpatient intravenous steroids*	Poorly bioavailable steroids†	IFX alone	Tofacitinib	Discussion with COVID-19 specialist‡
	Ambulatory intravenous steroids§	Intravenous steroids*+IFX	Ciclosporin	Colectomy	
Positive SARS-CoV-2 swab WITH symptoms or signs of COVID-19 pneumonia	Inpatient intravenous steroids*	Poorly bioavailable steroids†	IFX alone	Tofacitinib	Discussion with COVID-19 specialist‡
	Ambulatory intravenous steroids§	intravenous steroids*+IFX	Ciclosporin	Colectomy	

Green is considered appropriate, yellow uncertain and red inappropriate.

*Steroids, intravenous hydrocortisone 100 mg four times a day or intravenous methylprednisolone 60 mg daily as an inpatient.

†Budesonide MMX 9 mg/beclometasone 5 mg once daily orally as an inpatient; IFX (either 5 mg/kg or 10 mg/kg).

‡Discussion with appropriate COVID-19 specialist as per local availability.

§Intravenous methylprednisolone 60 mg daily as an outpatient.

IFX, infliximab; MMX, multimatrix.

### Rescue therapy

In patients meeting criteria for escalation of management at day 3, it was considered inappropriate to avoid rescue therapy by continuing monotherapy with intravenous corticosteroids, irrespective of COVID-19 status. Instead, the panellists deemed that following standard BSG guidance by initiating infliximab and continuing steroids was appropriate, whereas treatment with infliximab in conjunction with immediate steroid withdrawal was deemed uncertain. The BSG guidelines also recommend ciclosporin as an alternative rescue therapy. However, the RAND panel voted that ciclosporin, either with or without ongoing steroids, was inappropriate in all scenarios other than in patients with a negative SARS-CoV-2 swab in whom it was rated uncertain. Finally, colectomy without rescue therapy was deemed inappropriate in all the scenarios considered by the panel. However, once colectomy became necessary, for example where rescue therapy had failed or when complications had occurred, it was deemed inappropriate to delay surgery, even in patients with COVID-19 pneumonia (table 3).

**Table 3:** Appropriateness of treatment options in acute severe UC in the context of the COVID-19 pandemic: rescue therapy

	Rescue therapy				Failure of rescue therapy
Negative SARS-CoV-2 swab WITHOUT respiratory symptoms	Continue intravenous steroids alone	IFX +steroids	Intravenous ciclosporin +steroids	Colectomy	Delay surgery
		IFX, stop steroids	Intravenous ciclosporin, stop steroids	Discussion with COVID-19 specialist*	
Positive SARS-CoV-2 swab WITHOUT respiratory symptoms or signs of COVID-19 pneumonia	Continue intravenous steroids alone	IFX +steroids	Intravenous ciclosporin +steroids	Colectomy	Delay surgery
		IFX, stop steroids	Intravenous ciclosporin, stop steroids	Discussion with COVID-19 specialist*	
Positive SARS-CoV-2 swab WITH symptoms or signs of COVID-19 pneumonia	Continue intravenous steroids alone	IFX +steroids	Intravenous ciclosporin +steroids	Colectomy	Delay surgery
		IFX, stop steroids	Intravenous ciclosporin, stop steroids	Discussion with COVID-19 specialist*	

Green is considered appropriate, yellow uncertain and red inappropriate. Steroids, intravenous hydrocortisone 100 mg four times daily or intravenous methylprednisolone 60 mg daily as an inpatient; IFX (either 5 mg/kg or 10 mg/kg).

*Discussion with appropriate COVID-19 specialist as per local availability.

IFX, infliximab.

### Continuing medical therapy

The ongoing management of patients who had responded to intravenous corticosteroids and were ready for discharge on oral steroids was also considered. In patients with a negative SARS-CoV-2 swab, or with a positive swab but without signs or symptoms of pneumonia, steroid tapering over 6–8 weeks as per BSG guidance was deemed appropriate. However, in patients with COVID-19 pneumonia it was rated uncertain. Accelerated steroid withdrawal over 4–6 weeks was rated appropriate regardless of COVID-19 status. More rapid withdrawal over 4 weeks was deemed inappropriate except in patients with COVID-19 pneumonia, in whom it was rated uncertain. The use of poorly bioavailable oral steroids as an alternative to a standard steroid taper was rated as inappropriate in all scenarios (table 4).

**Table 4:** Appropriateness of treatment options in acute severe UC in the context of the COVID-19 pandemic: continuing medical therapy

	Continuing medical therapy*				
Negative SARS-CoV-2 swab WITHOUT respiratory symptoms	Standard steroid taper	Accelerated steroid taper <4 weeks	Thiopurine†	Ustekinumab†	Tofacitinib†
	Accelerated steroid taper 4–6 weeks	Poorly bioavailable steroids‡	Anti-TNF†	Vedolizumab†	Thromboprophylaxis§
Positive SARS-CoV-2 swab WITHOUT respiratory symptoms or signs of COVID-19 pneumonia	Standard steroid taper	Accelerated steroid taper <4 weeks	Thiopurine†	Ustekinumab†	Tofacitinib†
	Accelerated steroid taper 4–6 weeks	Poorly bioavailable steroids‡	Anti-TNF†	Vedolizumab†	Thromboprophylaxis§
Positive SARS-CoV-2 swab WITH symptoms or signs of COVID-19 pneumonia	Standard steroid taper	Accelerated steroid taper <4 weeks	Thiopurine†	Ustekinumab†	Tofacitinib†
	Accelerated steroid taper 4–6 weeks	Poorly bioavailable steroids‡	Anti-TNF†	Vedolizumab†	Thromboprophylaxis§

Green is considered appropriate, yellow uncertain and red inappropriate.

*Patient has responded to intravenous steroid therapy.

†Steroid taper and start additional therapy at or soon after discharge.

‡Switch from corticosteroids to budesonide MMX 9 mg daily/beclometasone 5 mg daily.

§Continue for a period after discharge.

MMX, multimatrix; TNF, tumour necrosis factor.

Initiation of additional therapy prior to or soon after discharge to prevent relapse was also considered. Following BSG guidance by initiating a thiopurine was rated uncertain in SARS-CoV-2 swab-negative patients and inappropriate in swab-positive patients. Use of biological therapy (anti-tumour necrosis factor (TNF), ustekinumab or vedolizumab) was deemed appropriate in swab-negative patients. In all other patients, the appropriateness of biological therapy was uncertain, except for anti-TNF therapy in patients with a positive swab but without pneumonia in whom treatment was rated as appropriate. Tofacitinib was generally rated as inappropriate except in swab-negative patients in whom it was rated uncertain.

Finally, panellists were asked whether patients should be discharged with a period of ongoing prophylactic anticoagulation. This was deemed appropriate in patients who had a positive SARS-CoV-2 swab regardless of whether they had pneumonia but was rated uncertain in those who had negative swabs.

## Discussion

### General considerations

The recent International Organisation For the Study of Inflammatory Bowel Disease RAND appropriateness panel addressing the use of medications to treat IBD in the COVID-19 era did not specifically address the management of patients with ASUC.^7^ To date, there has been no consensus on how to manage this condition during the COVID-19 pandemic; in the context of a limited, although rapidly evolving evidence base, this is perhaps unsurprising.^17^ Thus, there is an urgent need for guidance on how best to manage ASUC in the current setting. Several areas need consideration in this regard including: the effect of SARS-CoV-2 on the activity and course of IBD; the effect of IBD and its activity on the risk of being infected with SARS-CoV-2 and the progression to COVID-19; the interaction of SARS-CoV-2/COVID-19 with the drugs used to treat IBD; and the possible effects of treatments for COVID-19 on IBD.

SARS-CoV-2 is found in the gut and RNA is measurable in the stool significantly longer than in serum or respiratory samples^18^ although the significance of this is unclear. The effects of the virus on the intestinal mucosa remain undefined, as does its interaction with inflamed tissue.^19^ Gastrointestinal symptoms including diarrhoea occur in around 30% of patients and have been associated with worse outcome,^20 21^ and a single report describes a possible case of COVID-19 colitis.^22^

Currently, it is not clear whether IBD-specific factors lead to worse outcomes in patients who develop COVID-19. In the Italian series of 79 patients with IBD and COVID-19, active disease was associated with the risk of COVID-19 pneumonia even after controlling for other risk factors.^12^ Furthermore, active IBD was also significantly associated with increased hospitalisation, the need for respiratory support and death. In contrast, in Bergamo, Northern Italy, an observational study reported no cases of COVID-19 in 522 patients with IBD.^23^ While there are data that suggest that active IBD increases the risk of some viral infections,^24^ it is difficult to draw firm conclusions with regard to SARS-CoV-2 infection given the limited data available.

Of concern to most clinicians caring for patients with IBD is the possible risk of the drugs used to manage ASUC in the context of the COVID-19 pandemic. Intravenous corticosteroids remain the most widely used induction therapy in ASUC,^25^ but it is uncertain how they may influence outcome in patients with SARS-CoV-2 infection and COVID-19. Corticosteroids are known to increase the risk of sepsis and respiratory tract infections and may also increase viral replication and susceptibility to SARS-CoV-2.^26 27^ There is also evidence that steroids may increase morbidity and/or mortality from some respiratory viruses such as influenza, Middle East respiratory syndrome and SARS-CoV,^26 28–30^ although steroids have an established role in the management of ARDS.^31^ Beyond corticosteroids, immunomodulators such as thiopurines, biologics and tofacitinib are frequently used at various stages of the management of ASUC and there is also a lack of data regarding their safety in the context of the SARS-CoV-2 pandemic. Finally, it is important to consider the possible effects of drugs used to manage COVID-19 on IBD. For example, interleukin 6 inhibitors are being tested in patients with COVID-19 (ClinicalTrials.gov Identifier: NCT04315298) but have been associated with intestinal perforation in IBD.

We used an established methodology, a RAND appropriateness panel, to produce guidance in this challenging clinical area. Regarding initial management, there was agreement that all patients with ASUC should be managed as inpatients. Ambulatory care was considered inappropriate, since patients with ASUC need regular monitoring and involvement of a multidisciplinary team, this type of complex care being difficult to deliver in the outpatient setting. While there was some support for ambulatory management to avoid patients being admitted, thereby decreasing the risk of nosocomial acquisition of SARS-CoV-2, the risks of managing ASUC as an outpatient were considered to outweigh this possible benefit. Furthermore, in scenarios in which patients had confirmed SARS-CoV-2 infection, no such benefit existed. Nevertheless, in view of the acknowledged risk of contracting SARS-CoV-2 infection in hospital, it is perhaps unsurprising that the panel considered it appropriate to isolate patients with ASUC in a side room wherever possible.

The panel deemed it uncertain whether a CT chest should be performed in all patients on admission. While a CT chest is more sensitive than a chest X-ray (CXR) in detecting signs of early or limited infection, the COVID-19 specialists advised that a CXR would suffice in asymptomatic patients on admission. However, the Royal College of Radiologists has advised a low-dose CT chest should be performed in patients who are having a CT abdomen as part of the investigation of an abdominal emergency.^32–34^

It was considered appropriate to involve a COVID-19 specialist in all scenarios in the presence of a positive SARS-CoV-2 swab, regardless of signs or symptoms of COVID-19 pneumonia. The panel was uncertain whether this was required in patients with a negative SARS-CoV-2 swab who required rescue therapy. During the meeting, concern was expressed by some panellists about the possible effects of corticosteroids and rescue therapies on SARS-CoV-2 infection and COVID-19 pneumonia driving the need to seek clarification from COVID-19 experts and highlighting the need for further research.

### First-line therapy

It was considered appropriate to follow the BSG guidelines on the initial management of ASUC in patients without signs or symptoms of COVID-19, regardless of SARS-CoV-2 swab results. Only in patients with COVID-19 pneumonia was there uncertainty among the panel regarding the appropriateness of conventional therapy with intravenous corticosteroids, largely driven by concerns of possible harm. However, it should be noted that in this challenging condition in which there is scant experience and almost no published data in relation to COVID-19, of all suggested treatments, intravenous corticosteroids were given the highest median score by the panel. Regarding the ongoing uncertainty about the benefits or harms of corticosteroids in patients with COVID-19 pneumonia and the inconclusive data emerging from the current coronavirus pandemic, the results of the adaptive trial, RECOVERY, which includes a dexamethasone arm, are eagerly awaited.^1^ Nevertheless, leaving ASUC untreated is associated with a high risk of death, mortality being at least 24% in the days before the use of corticosteroids.^25^ The expert advisers supported the WHO position that steroid use should not be avoided because of theoretical risks in patients with COVID-19.^35^

The panel was uncertain whether infliximab, without concurrent corticosteroids, should be used as a first-line therapy in patients who are SARS-CoV-2 positive, regardless of whether they had COVID-19. As with corticosteroids, the risk of anti-TNF in the context of the pandemic is unknown. In addition, there is no high-quality evidence for infliximab in ASUC other than as a rescue therapy following corticosteroid failure. Anti-TNF agents are known to increase the risk of respiratory tract and other opportunistic infections,^36^ particularly when used in association with thiopurines and corticosteroids.^37^ However, anti-TNF therapies are currently being evaluated in clinical trials^38^ as a potential treatment for COVID-19-induced cytokine storm.^39 40^ In view of the uncertainty of the effects of corticosteroids and infliximab on SARS-CoV-2 infection, it was considered appropriate that all patients with a positive swab should be discussed with a COVID-19 specialist to guide decision-making.

### Rescue therapy

Up to half of patients with ASUC fail first-line medical therapy with corticosteroids.^6^ In all scenarios, it was considered inappropriate to continue this treatment alone in the face of non-response at day 3, consistent with current BSG guidelines.^6^ Similarly, in line with BSG guidance, it was considered appropriate to commence infliximab while continuing corticosteroids regardless of SARS-CoV-2 status. Discontinuation of corticosteroids at the point of commencing infliximab rescue therapy was considered of uncertain appropriateness across all scenarios, as it may result in worsening colitis, while acknowledging the potential risks of combining the two drugs. Ciclosporin rescue therapy was generally considered inappropriate, due in part to concerns about the risks of drug-induced nephrotoxicity given the frequency of acute kidney injury in SARS-CoV-2 infection.^41^ In addition, the infusion regimen requires frequent healthcare worker–patient contact which could, in theory, increase the risk of transmission. The panel did not explore its use in settings in which infliximab may be relatively contraindicated, such as previous loss of response to infliximab, drug immunogenicity or when relevant comorbidities exist, such as multiple sclerosis. Similarly, the panel did not specifically address the question of whether infliximab was used as a monotherapy or in combination with an immunomodulator.

There is little evidence regarding the risks of surgical management in patients with COVID-19. Preliminary data demonstrate a substantial increase in morbidity and mortality among patients infected with SARS-CoV-2 undergoing surgery. In one report, 34 patients underwent elective surgery in Wuhan, China, with all developing COVID-19 pneumonia, 7 of whom (20%) died.^9^ Accordingly, the risks of surgery drove the rating of colectomy as first-line therapy or as an alternative to rescue therapy, as being inappropriate. However, in patients failing medical therapy, there was consensus that delaying surgery would be inappropriate.

### Continuing medical therapy

The BSG IBD guidelines recommend corticosteroid tapering over 6–8 weeks which was considered appropriate by the panel, except in the context of COVID-19 pneumonia where an accelerated taper over 4–6 weeks was considered appropriate instead. A more accelerated taper, over fewer than 4 weeks, was generally deemed inappropriate due to the high risk of relapse in this cohort.^6^ Regarding initiation of maintenance therapy either before or shortly after discharge from hospital, it was considered appropriate to start anti-TNF, vedolizumab or ustekinumab in patients with negative swabs. However, in scenarios in which patients had positive swabs, with or without evidence of COVID-19 pneumonia, there was uncertainty about the risk:benefit ratio of biological therapy, driven by the lack of evidence. Thus, biological use in this situation was deemed uncertain in nearly all scenarios.

Thiopurines and tofacitinib were not considered appropriate at any stage during the scenarios. This is despite the BSG recommendation that thiopurines should be initiated at or soon after discharge following successful treatment of ASUC.^6^ Azathioprine therapy was in part considered inappropriate due to possible side effects such as pancreatitis, which could result in readmission to hospital, and drug hypersensitivity, which can manifest as a flu-like syndrome which may potentially be confused with COVID-19.^42^ Azathioprine can also induce significant lymphopaenia^42^ which may mimic the lymphopaenia seen in SARS-CoV-2 infection. How this affects outcome of COVID-19 is unclear; some reports even suggest a theoretical benefit of thiopurines.^43 44^ The additional monitoring required when azathioprine is initiated may also be a challenge with COVID-19-related service reconfiguration and antecedent risks of SARS-CoV-2 acquisition posed by the requirement for face-to-face contact from laboratory monitoring.

Tofacitinib is a non-selective Janus kinase inhibitor which is associated with herpes zoster viral reactivation and, like COVID-19, is also associated with an increased risk of deep vein thrombosis.^45^ There is also very limited evidence for its use in the setting of ASUC.^46^ For these reasons, the panel considered its use inappropriate in nearly all settings although it was noted that its rapid offset of action could be of theoretical benefit.

### Anticoagulation

Prophylactic anticoagulation was considered appropriate beyond discharge among patients with a positive SARS-CoV-2 swab, although this strategy was deemed uncertain in people with negative swabs. Like ASUC, COVID-19 is strongly linked to a hypercoagulable state with substantially increased risk of microthrombi and venous thromboembolism (VTE).^47^ It is notable that the British Thoracic Society recommends doubling the dose of anticoagulation and/or prescribing VTE prophylaxis (low-molecular-weight heparin or direct oral anticoagulant) for up to 4 weeks following discharge in high-risk patients with COVID-19.^48^

### Strengths and limitations

The strengths of our study include the inclusion of a diverse group of IBD experts drawn from a wide range of UK centres as well as non-gastroenterology specialists with experience in managing patients with COVID-19. In addition, we used the RAND methodology which is a validated technique to guide decision-making in the absence of a robust evidence base. It is not necessarily an attempt to reach consensus but rather to guide clinicians as to the appropriateness or inappropriateness of interventions, while accepting that uncertainty is also a valid outcome, which was highly appropriate in this setting. It was impossible for our scenarios to encompass fully all cases encountered in clinical practice. We, therefore, focused on principles that may help to guide decision-making in most cases of ASUC in the context of COVID-19. We appreciate that by doing so, this guidance may not be directly applicable to more nuanced cases where decision-making may be influenced by a myriad of factors. Nor was every aspect of care considered; for example, the question of repeating testing for *Clostridium difficile* prior to colectomy in view of higher exposure to antibiotics in the COVID-19 era was not addressed. The outcomes should, therefore, be considered an adjunct to multidisciplinary decision-making rather than a replacement. Finally, knowledge within the field remains fast moving such that it will be important to stay abreast of new developments as they arise.

### Implications and concluding remarks

By combining clinical expertise from the BSG CRG and IBD Section Committee in conjunction with other medical and surgical IBD and COVID-19 experts, we have provided guidance to clinicians regarding the appropriate management of ASUC during the COVID-19 pandemic, highlighting where current BSG guidance may need adaptation. Population-based studies are needed to clarify the risks and benefits of interventions used in the management of ASUC during the pandemic. Until then, we consider the results of the panel, which largely support following the well-established and evidence-based BSG guideline, will help guide clinicians in this challenging and evolving area.
