# Supplementary material for: Adaptations to the British Society of Gastroenterology guidelines on the management of acute severe UC in the context of the COVID-19 pandemic: a RAND appropriateness panel
Source: Gut. 2020 Jun 8;69(10):1769–77. doi: 10.1136/gutjnl-2020-321927 (PMC7299646; doi:10.1136/gutjnl-2020-321927)
Supplement: Supplementary data [file gutjnl-2020-321927supp001.pdf]

Supplementary Table 1: RAND Panel members

RAND Panellists	Affiliation
**Shahida Din***	Western General Hospital, Edinburgh
**Alex Kent**	King's College Hospital NHS Foundation Trust, London
**Richard Pollok***	St George's University Hospitals NHS Foundation Trust, London
**Nick Kennedy^**	Royal Devon and Exeter NHS Foundation Trust, Exeter
**Robin Dart**	Royal Free London NHS Foundation Trust, London
**Daniel Gaya***	Glasgow Royal Infirmary, NHS Greater Glasgow and Clyde
**Ailsa Hart^**	St Mark's Hospital North West University Healthcare NHS Trust, London
**Chris Lamb***	Newcastle upon Tyne Hospitals NHS Foundation Trust
**Jimmy Limdi**	The Pennine Acute Hospitals NHS Trust, Manchester
**James Lindsay^**	The Royal London Hospital, Barts Health NHS Trust, London
**Chris Probert**	University of Liverpool
**Tim Raine^**	Cambridge University Hospitals NHS Foundation Trust
**Christian Selinger***	Leeds Teaching Hospitals NHS Trust
**Shaji Sebastian*^**	Hull University Teaching Hospitals NHS Trust
**Lisa Younge**	St Mark's Hospital, North West University Healthcare NHS Trust, London

*Denotes British Society of Gastroenterology Inflammatory Bowel Disease Section Committee member
^Denotes British Society of Gastroenterology Inflammatory Bowel Disease Clinical Research Group member
