# Supplementary material for: Adaptations to the British Society of Gastroenterology guidelines on the management of acute severe UC in the context of the COVID-19 pandemic: a RAND appropriateness panel
Source: Gut. 2020 Jun 8;69(10):1769–77. doi: 10.1136/gutjnl-2020-321927 (PMC7299646; doi:10.1136/gutjnl-2020-321927)
Supplement: Supplementary data [file gutjnl-2020-321927supp002.pdf]

Supplementary Table 2. Final Assessment of Statements by RAND Panel: Appropriateness of management in acute severe ulcerative colitis (ASUC) in the context of COVID19

91 Statements	Median	Disagreement Index	Standard Deviation	Category
**Admission**				
**All patients admitted with ASUC**				
Perform a SARS-CoV-2 swab on admission	8	0.13*	0.51	Appropriate
Perform a CT chest within 24 hours of admission	4	0.52	1.39	Uncertain
Perform a CT abdomen, in addition to AXR, within 24 hours of admission	3	0.45	2.09	Inappropriate
Isolate them in a side room throughout their admission regardless of COVID status	8	0.23	1.21	Appropriate
**Perform a flexible sigmoidoscopy**				
Within 24 hours of admission in all patients admitted with ASUC (as per BSG guidance)	8	0.00	1.96	Appropriate
In all patients failing IV corticosteroids who have not had a flexible sigmoidoscopy on admission	9	0.13*	0.64	Appropriate
In all patients failing intravenous corticosteroid therapy who have already had a flexible sigmoidoscopy on admission	3	0.00	0.88	Inappropriate
In all patients being referred for colectomy who have not had a flexible sigmoidoscopy on admission, to confirm the diagnosis prior to surgery (excluding patients who have toxic megacolon or perforation)	8	0.23*	0.85	Appropriate
In all patients being referred for colectomy who have already had a flexible sigmoidoscopy on admission, to assess the degree of ongoing inflammation (excluding patients who have toxic megacolon or perforation)	2	0.16	0.94	Inappropriate
**First line medical therapy**				
**Negative swab and no respiratory symptoms**				
Follow standard BSG guidelines and start intravenous hydrocortisone/methylprednisolone	9	0.10	0.92	Appropriate

Start IV methylprednisolone 60 mg daily as an outpatient (with daily specialist review, once daily observations and access to x-ray and bloods as required)	3	0.30	2.18	Inappropriate
Start budesonide MMX 9 mg or beclometasone 5 mg daily PO (as an inpatient)	1	0.13*	0.74	Inappropriate
Start IV steroids concurrently with infliximab	3	0.13	2.06	Inappropriate
Start infliximab without steroids	3	0.33	1.83	Inappropriate
Start ciclosporin monotherapy as a bridge to another therapy	2	0.16	1.41	Inappropriate
Start tofacitinib 10 mg bd	2	0.13	1.64	Inappropriate
Colectomy	1	0.10*	0.74	Inappropriate
Discuss with COVID-19 specialist	3	0.16	0.92	Inappropriate
**Positive swab but no symptoms or signs of COVID-19 pneumonia**				
Follow standard BSG guidelines and start intravenous hydrocortisone/methylprednisolone	7	0.13	0.80	Appropriate
Start IV methylprednisolone 60 mg daily as an outpatient (with daily specialist review, once daily observations and access to x-ray and bloods as required)	2	0.29	2.13	Inappropriate
Start budesonide MMX 9 mg or beclometasone 5 mg daily PO (as an inpatient)	1	0.10*	0.63	Inappropriate
Start IV steroids concurrently with infliximab	2	0.16	1.58	Inappropriate
Start infliximab without steroids	4	0.52	1.94	Uncertain
Start ciclosporin monotherapy as a bridge to another therapy	2	0.16	1.36	Inappropriate
Start tofacitinib 10 mg bd	1	0.13	1.10	Inappropriate
Colectomy	1	0.13*	0.64	Inappropriate
Discuss with COVID-19 specialist	7	0.13	1.41	Appropriate
**Positive swab with symptoms or signs of COVID-19 pneumonia**				
Follow standard BSG guidelines and start intravenous hydrocortisone/methylprednisolone	6	0.45	1.42	Uncertain
Start IV methylprednisolone 60 mg daily as an outpatient (with daily specialist review, once daily observations and access to x-ray and bloods as required)	1	0.00*	0.72	Inappropriate

Start budesonide MMX 9 mg or beclometasone 5 mg daily PO (as an inpatient)	1	0.00	2.07	Inappropriate
Start IV steroids concurrently with infliximab	2	0.29	1.98	Inappropriate
Start infliximab without steroids	5	0.49	1.95	Uncertain
Start ciclosporin monotherapy as a bridge to another therapy	1	0.13	1.39	Inappropriate
Start tofacitinib 10 mg bd	1	0.10*	0.49	Inappropriate
Colectomy	1	0.13*	0.74	Inappropriate
Discuss with COVID-19 specialist	9	0.00*	0.59	Appropriate
**Rescue Therapy**				
Repeat a SARS-CoV-2 swab in patients with a negative first swab	7	0.22	2.03	Appropriate
**Negative swab and no respiratory symptoms**				
Continue intravenous steroids alone	1	0.00*	0.74	Inappropriate
Start infliximab and continue steroids	8	0.23*	0.85	Appropriate
Start infliximab and discontinue steroids	4	0.52	1.59	Uncertain
Start intravenous ciclosporin therapy with steroids (unless failed thiopurine)	5	0.95	2.02	Uncertain
Start intravenous ciclosporin and discontinue steroids (unless failed thiopurine)	3	0.22	1.59	Inappropriate
Colectomy	3	0.33	1.79	Inappropriate
Discuss with COVID-19 specialist	5	0.95	1.85	Uncertain
**Positive swab but no symptoms or signs of COVID-19 pneumonia**				
Continue intravenous steroids alone	1	0.00*	0.62	Inappropriate
Start infliximab therapy with steroids	7	0.16	1.05	Appropriate
Start infliximab and discontinue steroids	6	0.52	1.59	Uncertain
Start intravenous ciclosporin therapy with steroids (unless failed thiopurine)	3	0.22	1.29	Inappropriate
Start intravenous ciclosporin and discontinue steroids (unless failed thiopurine)	3	0.16	1.25	Inappropriate
Colectomy	2	0.33	1.74	Inappropriate
Discuss with COVID-19 specialist	8	0.26*	0.88	Appropriate
**Positive swab with symptoms or signs of COVID-19 pneumonia**				
Continue intravenous steroids alone	1	0.00	0.92	Inappropriate
Start infliximab with steroids	7	0.17	1.06	Appropriate
Start infliximab and discontinue steroids	5	0.52	1.54	Uncertain

Start intravenous ciclosporin therapy with steroids (unless failed thiopurine)	2	0.27	1.46	Inappropriate
Start intravenous ciclosporin and discontinue steroids (unless failed thiopurine)	2	0.29	2.07	Inappropriate
Colectomy	2	0.29	1.64	Inappropriate
Discuss with COVID-19 specialist	9	0.10*	0.74	Appropriate
**Continuing medical therapy**				
**Negative swab and no respiratory symptoms**				
Follow standard BSG guidelines for tapering oral steroids over 6-8 weeks	8	0.16	1.06	Appropriate
Use an accelerated steroid taper over 4-6 weeks	7	0.13	1.03	Appropriate
Use an accelerated steroid taper over fewer than 4 weeks	3	0.30	1.55	Inappropriate
Switch to budesonide MMX 9 mg or beclomethasone 5 mg daily PO	3	0.16	0.83	Inappropriate
Taper steroids and follow standard BSG guidelines initiating thiopurine therapy at or soon after discharge	4	0.45	1.77	Uncertain
Taper steroids and initiate anti-TNF therapy at or soon after discharge	7	0.00	0.64	Appropriate
Taper steroids and initiate ustekinumab at or soon after discharge	7	0.00	1.76	Appropriate
Taper steroids and initiate vedolizumab at or soon after discharge	7	0.22	1.72	Appropriate
Taper steroids and initiate tofacitinib at or soon after discharge	4	0.86	1.92	Uncertain
Continue prophylactic anticoagulation for a period after discharge	5	0.95	1.98	Uncertain
**Positive swab but no symptoms or signs of COVID-19 pneumonia**				
Follow standard BSG guidelines for tapering oral steroids over 6-8 weeks	7	0.45	1.58	Appropriate
Use an accelerated steroid taper over 4-6 weeks	7	0.16	0.70	Appropriate
Use an accelerated steroid taper over fewer than 4 weeks	3	0.45	1.82	Inappropriate
Switch to budesonide MMX 9 mg or beclomethasone 5 mg daily PO	3	0.16	1.35	Inappropriate
Taper steroids and follow standard BSG guidelines initiating thiopurine therapy at or soon after discharge (but within the period of potential ongoing SARS-CoV-2 infection)	3	0.16	1.49	Inappropriate

Taper steroids and initiate anti-TNF therapy at or soon after discharge (but within the period of potential ongoing SARS-CoV-2 infection)	7	0.22	1.21	Appropriate
Taper steroids and initiate ustekinumab at or soon after discharge (but within the period of potential ongoing SARS-CoV-2 infection)	6	0.52	1.71	Uncertain
Taper steroids and initiate vedolizumab at or soon after discharge (but within the period of potential ongoing SARS-CoV-2 infection)	6	0.52	1.73	Uncertain
Taper steroids and initiate tofacitinib at or soon after discharge (but within the period of potential ongoing SARS-CoV-2 infection)	3	0.00	0.83	Inappropriate
Continue prophylactic anticoagulation for a period after discharge	7	0.00	1.31	Appropriate
**Positive swab with symptoms or signs of COVID-19 pneumonia**				
Follow standard BSG guidelines for tapering oral steroids over 6-8 weeks	6	0.84	1.92	Uncertain
Use an accelerated steroid over 4-6 weeks	7	0.13	0.88	Appropriate
Use an accelerated steroid taper over fewer than 4 weeks	4	0.52	2.15	Uncertain
Switch to budesonide MMX 9 mg or beclomethasone 5 mg daily PO	2	0.27	1.55	Inappropriate
Taper steroids and follow standard BSG guidelines initiating thiopurine therapy at or soon after discharge (but within the period of potential ongoing SARS-CoV-2 infection)	3	0.16	1.29	Inappropriate
Taper steroids and initiate anti-TNF therapy at or soon after discharge (but within the period of potential ongoing SARS-CoV2 infection)	6	0.45	1.56	Uncertain
Taper steroids and initiate ustekinumab at or soon after discharge (but within the period of potential ongoing SARS-CoV-2 infection)	6	0.49	1.94	Uncertain
Taper steroids and initiate vedolizumab at or soon after discharge (but within the period of potential ongoing SARS-CoV-2 infection)	5	0.52	1.68	Uncertain
Taper steroids and initiate tofacitinib at or soon after discharge (but within the period of potential ongoing SARS-CoV-2	3	0.27	1.30	Inappropriate

infection)				
Continue prophylactic anticoagulation for a period after discharge	8	0.13*	0.83	Appropriate
**Surgery**				
In patients with a SARS-CoV-2 positive swab who have failed medical therapy, surgery should be delayed	3	0.17	1.29	Inappropriate
Patients with a negative swab on admission should have a repeat swab	8	0.16	1.51	Appropriate
Patients should have a CT chest prior to surgery regardless of swab status, respiratory symptoms examination findings and observations	8	0.16	0.92	Appropriate

* Denotes questions where all panelists voted the same appropriateness category as the final outcome category (i.e. level of appropriateness was agreed unanimously)
